# Dissociable Neural Responses to Monetary and Social Gain and Loss in Women With Major Depressive Disorder

**DOI:** 10.3389/fnbeh.2019.00149

**Published:** 2019-07-11

**Authors:** Anjali Sankar, Ashley A. Yttredahl, Elizabeth W. Fourcade, Brian J. Mickey, Tiffany M. Love, Scott A. Langenecker, David T. Hsu

**Affiliations:** ^1^Department of Psychiatry, Yale School of Medicine, New Haven, NY, United States; ^2^Department of Psychiatry and Behavioral Health, Stony Brook University, Stony Brook, NY, United States; ^3^Department of Psychology, Stony Brook University, Stony Brook, NY, United States; ^4^Department of Psychiatry, Allegheny Health Network, Pittsburgh, PA, United States; ^5^Department of Psychiatry, The University of Utah, Salt Lake City, UT, United States; ^6^Department of Psychiatry, University of Michigan, Ann Arbor, MI, United States

**Keywords:** major depression, women, functional magnetic resonance imaging, social feedback, monetary incentive delay task, reward and loss

## Abstract

Neuroimaging studies have revealed aberrant reward and loss processing in patients with major depressive disorder (MDD). While most studies use monetary stimuli to study these processes, it is important to consider social stimuli given that the social environment plays a significant role in the development and maintenance of MDD. In the present study, we examined whether monetary gain/loss and social acceptance/rejection would elicit dissociable salience-related neural responses in women diagnosed with MDD compared to healthy control (HC) women. Twenty women diagnosed with MDD and 20 matched HC women performed the monetary incentive delay task (MID) and the social feedback task (SFT) during functional magnetic resonance imaging (fMRI). This study focused on women since women have a higher rate of MDD, higher frequency of relapse, and are more likely to develop MDD as a consequence of negative interpersonal relationships compared to men. We found that during the MID, HCs but not MDD patients demonstrated strong overlapping activations in the right anterior insula (AI) in response to both monetary gain and loss. During the SFT, MDD patients but not HCs showed overlapping activations in the AI in response to social acceptance and rejection. Our results may suggest a dissociation such that MDD patients show decreased sensitivity to monetary stimuli whether gain or loss, and increased sensitivity to social stimuli whether acceptance or rejection, although this will need to be verified in larger samples with direct comparisons between groups and stimuli. These data demonstrate distinct abnormalities in reward and loss processing that converge within the AI. Our findings also highlight the critical need to assess across both non-social and social domains when examining reward and loss systems in MDD to broaden our understanding of the disorder and identify novel targets for treatment.

## Introduction

Anhedonia, defined as the loss of interest in previously rewarding activities, is a core feature of major depressive disorder (MDD; American Psychiatric Association, 2013), yet it is not effectively managed with first-line antidepressant treatments (Shelton and Tomarken, 2001) and is generally associated with poor treatment outcomes (Spijker et al., 2001). The last decade has seen a preponderance of work on maladaptive neural responses to both reward and loss in MDD. Much of this research has focused on monetary reward and loss (Knutson et al., 2008; Pizzagalli et al., 2009; Smoski et al., 2009; Olino et al., 2011; Chandrasekhar Pammi et al., 2015; Ubl et al., 2015). However, MDD is often caused and maintained by maladaptive responses to *social* reward and loss, defined here as social acceptance and rejection, respectively. Social acceptance includes social support which has been shown to lessen the impact of stressors (Viswesvaran et al., 1999; Kaufman et al., 2004; Zimmer-Gembeck et al., 2007) and mitigates MDD symptoms (George et al., 1989). On the other hand, social rejection—when one is not wanted or liked—includes experiences such as parental rejection, peer victimization, and romantic rejection, all of which are known to precipitate and exacerbate MDD symptoms (Boyce et al., 1992; Rapee, 1997; Joiner and Coyne, 1999; Monroe et al., 1999; Kendler et al., 2003; Slavich et al., 2009; Copeland et al., 2013). Thus, the social environment plays an important role in the development and maintenance of MDD.

In healthy controls (HCs), a recent meta-analysis showed that both monetary and social reward anticipation engaged a common neural circuit encompassing the ventral striatum (nucleus accumbens, NAcc) and anterior insula (AI), along with the ventral tegmental and supplementary motor areas (Gu et al., 2019). The NAcc and the AI have also been shown to be engaged during monetary loss (Dugré et al., 2018; Oldham et al., 2018; Wilson et al., 2018) as well as during social loss (Eisenberger et al., 2003; Gunther Moor et al., 2010). The anterior cingulate cortex (ACC) is also implicated in the processing of monetary and social incentives (Rademacher et al., 2010; Dugré et al., 2018; Wilson et al., 2018), however there is considerable evidence for valence-dependent activations in the ACC with greater sensitivity to losses or reward reduction compared to gains (Bush et al., 2002; Gehring and Willoughby, 2002; Liu et al., 2011). Together, findings from HCs point to a core neural circuitry comprising the ventral striatum, the AI, and potentially the ACC, that is common to monetary and social reward and loss.

Emerging data from MDD studies suggest that abnormal neural responses to reward and loss in MDD depend on the type of stimuli (monetary or social) and particularly the salience associated with them. Studies using monetary incentives have shown reduced neural responsivity in the ventral striatum especially in the NAcc, and in the medial prefrontal cortex to monetary gain and loss in MDD (Steele et al., 2007; Pizzagalli et al., 2009; Stoy et al., 2012; Ubl et al., 2015). On the other hand, positive social feedback in MDD is associated with enhanced neural responsivity in the amygdala (Davey et al., 2011) and social rejection in MDD is associated with enhanced neural responsivity in the NAcc (Silk et al., 2014), AI and the amygdala (Silk et al., 2014; Kumar et al., 2017; Yttredahl et al., 2018). Consistent with the neural responses, behavioral responses also are heightened in response to social acceptance and rejection to MDD (Hsu et al., 2015; Yttredahl et al., 2018), indicating that social feedback may be especially salient in MDD. Thus, it is possible that MDD is characterized by hypo- and hyper-neural and behavioral responsivity to monetary and social stimuli, respectively. However, unlike neuroimaging studies in HCs that compared neural responses to monetary vs. social stimuli in HCs (e.g., Izuma et al., 2008; Rademacher et al., 2010; Lin et al., 2011; Xie et al., 2014), no study has examined if salience-related neural responses are differently represented in MDD based on the type of incentive stimuli.

We focused on the role of the AI and NAcc as *a priori* regions of interest (ROIs) because both are engaged during processing motivationally salient stimuli (Zink et al., 2003; Cooper and Knutson, 2008; Menon and Uddin, 2010), and have shown activations in response to both monetary and social stimuli during both reward and loss in MDD and HC (Elliott et al., 2000; Levita et al., 2009; Rademacher et al., 2010; Liu et al., 2011; Hsu et al., 2013, 2015; Zhang et al., 2013; Floresco, 2015; Achterberg et al., 2016; Dalgleish et al., 2017; Perini et al., 2018). Although the ACC is involved in processing monetary and social incentives (Rademacher et al., 2010; Dugré et al., 2018; Wilson et al., 2018), it appears to be involved mainly in processing monetary or social loss (Bush et al., 2002; Gehring and Willoughby, 2002; Liu et al., 2011; Silk et al., 2014; Yttredahl et al., 2018).

Thus, the goal of the present study was to systematically examine salience-related AI and NAcc activation during monetary and social reward and loss in MDD patients and HCs, in which each participant performed a monetary and social task during the same functional magnetic resonance imaging (fMRI) scan session. For the purpose of this study, the primary analysis for the monetary task focused on a subset of “certain trials” (which cued a guaranteed reward or a loss), as opposed to uncertain trials, as they indicated a known outcome, comparable to the known outcome of receiving acceptance or rejection feedback from the social task used in this study. Although outcomes from uncertain trials also engage the AI and the NAcc, these regions are also seen engaged during certain outcomes. For instance, AI activation was observed during decision making even in the presence of certain outcomes (Feinstein et al., 2006), and the NAcc was shown to be engaged even during certain rewards (Cooper and Knutson, 2008).

We tested MDD women and matched HC women (ages 18–55 years). Compared to men, women have higher rates of MDD, a more chronic course of the disorder (Essau et al., 2010), younger age of onset (Marcus et al., 2005), and more frequent relapse episodes (Oquendo et al., 2013). Furthermore, negative interpersonal relationships have been shown to be more predictive of MDD in women compared to men (Kendler et al., 2005; Kendler and Gardner, 2014). Thus, social stimuli may be more salient in eliciting the neural responses that are critical to understanding the pathophysiology of MDD in women. Since social stimuli are notably salient to MDD patients, we hypothesized heightened activations during social acceptance and rejection in MDD relative to HCs in these regions. Demonstrating this distinction would be critical in understanding the nature, function, and clinical implications of reward-related abnormalities, ultimately leading to novel treatment strategies in MDD (Stoy et al., 2012; Zhang et al., 2013).

### Materials and Methods

#### Participants

Twenty women with MDD (ages 18–55 years; mean age ± standard deviation: 30.00 ± 10.84 years) and 20 HC women (ages 18–53 years; 30.25 ± 10.99 years), matched for age, sexual orientation, ethnicity, and relationship status were recruited from the community through local advertisements. Demographic and clinical characteristics are presented in Table 1. MDD patients were assessed for current depressive episode and HCs were screened for current or past history of psychiatric disorders using the Mini-International Neuropsychiatric Interview (MINI; Sheehan et al., 2006). Patient scores ranged from 10 to 21 (mild to moderate; Zimmerman et al., 2013) on the 17-item Hamilton Depression Rating Scale (HAM-D 17; Hamilton, 1960) and had a mean score of 14.88 ± 2.95. All patients had a primary diagnosis of MDD. As expected, most patients (*n* = 17) reported symptoms of anxiety, however only one met criteria for current comorbid social anxiety disorder (DSM-IV criteria assessed using the MINI). Four MDD patients were taking antidepressants during the course of the study but were on stable doses for at least 4 weeks prior to study recruitment. All other participants were free of psychotropic substances for at least 2 months, regular tobacco use, history of DSM-IV alcohol or drug dependence within the past 5 years, or alcohol or drug abuse in the past 2 years. All protocols were approved by the University of Michigan Medical School Institutional Review Board, and written informed consent was obtained from all participants.

**Table 1:** Demographics and clinical characteristics.

	MDD Patients	Healthy Controls
Participants	20 women	20 women
Age	30.00 (10.84)	30.25 (10.99)
HAM-D	14.88 (2.95)	NA
Age of MDD onset	18.38 (7.37)	NA
Sexual Orientation (Heterosexual/Homosexual/Bisexual)	16/0/4	19/1/0
**Ethnicity**	
Asian	0	2
Caucasian	15	14
Black or African American	4	3
Mixed	1	1
**Relationship Status**	
Single	11	12
In a relationship	6	5
Married	3	3

*Abbreviations: MDD: major depressive disorder; HAM-D: 17-item Hamilton Depression Rating Scale. Mean values and standard deviations in parentheses are presented for Age, HAM-D, and Age of MDD onset. Sexual orientation, ethnicity, and relationship status are presented as the number of participants*.

#### Monetary Incentive Delay Task

**Figure 1:**
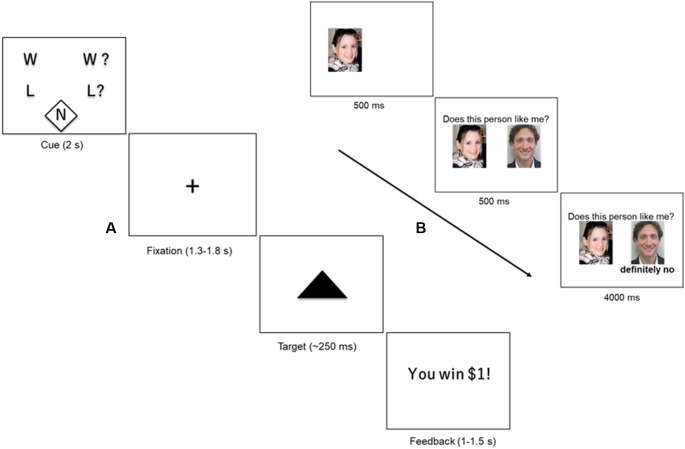
**(A)** Monetary Incentive Delay Task (MID). Participants were presented with one of five cues (2 s) indicating the type of trial: certain win (W), uncertain win (W?), certain loss (L) uncertain loss (L?), or neutral (N). The cue was followed by a crosshair fixation of variable display period (1.3–1.8 s) followed by a short presentation of a target (~250 ms). Participants were instructed to hit the target using a button-press response box. At the end of the trial, the participant received feedback about the outcome of the trial (1–1.5 s), followed by a variable inter-trial interval (ITI) of 2–6 s. **(B)** Social Feedback Task (SFT). Each trial of the SFT begins with a picture of the subject, displayed for 500 ms, followed by a picture of a highly rated profile (500 ms) along with his/her rating of the subject (“feedback”; 4,000 ms). A rejection trial is presented. Figure adapted from our previous study (Yttredahl et al., 2018).

We used a version of the Monetary Incentive Delay Task (MID) described in Warthen et al. (2019). Briefly, in this event-related paradigm (Figure 1A), participants saw one of five cues (2 s), based on the trial type, followed by a delay phase indicated by a fixation cross (1.3–1.8 s). The delay phase was followed by brief presentation of a solid black triangle (~250 ms). Participants were instructed to hit the target with a button press as quickly as possible, irrespective of the trial type. We varied the presentation time of the target dynamically based on participant’s performance without their knowledge to ensure an average hit rate of about 60%. Following presentation of the brief target, subjects received feedback on whether they won or lost money based on the trial type (randomized 1–1.5 s). The feedback phase was followed by a variable inter-trial interval (ITI) of 2–6 s.

Trial type varied across two dimensions: valence (reward or loss) and certainty (certain or uncertain). Certain and uncertain trials have previously been described as “low-salience” and “high-salience” trials, respectively (Mickey et al., 2016), however, in the present study, we use the terms “certain” and “uncertain” to avoid confusion with discussions of saliency in other contexts in this manuscript. Valence was manipulated by varying the incentive outcome (positive outcomes = reward; and negative outcome = loss). Certainty was manipulated by varying the certainty associated with the outcome. For instance, in uncertain trials, participants were instructed to respond when the target appeared on screen for a chance to win money ($1; reward trials) or to avoid losing money ($1; loss trials). In other words, the outcome was uncertain (Uncertain Wins: UW; Uncertain Losses: UL). In certain trials, participants were instructed to respond to the target but were told that the outcome was certain and that their response did not have an impact on whether they won or lost $1 (Certain Wins: CW; Certain Losses: CL). The neutral trials ($0; Neu) did not have any money at stake, but participants were nevertheless instructed to respond to the target. The five trial types (CW, CL, UW, UL and Neu) were presented 10 times in a pre-defined pseudorandomized order during each run. There was a total of two runs, and a single run consisted of 10 presentations of each trial type (50 presentations in total). Each run lasted approximately 8 min and 30 s plus approximately 30 shim time between runs.

#### Social Feedback Task

Data collected from our previous study (Yttredahl et al., 2018) using the SFT for fMRI was used in the present study. Several days before the fMRI scan (17.88 ± 9.33 days, range 4–46 days), participants viewed fictitious dating profiles of preferred-sex individuals (potential partners) and were asked to rate these profiles based on how much they liked the potential partner and how much they thought the potential partner would like them back. There was no significant association between the number of days elapsed between profile ratings and behavioral measures collected on the day of the scan (self-esteem, desire to socialize, feeling happy and accepted, and feeling sad and rejected; Pearson’s correlation coefficient *r*’s > 0.17, *p*’s > 0.30). In addition, there was no difference in the number of days elapsed between ratings and the day of the scan between HCs and MDD (2-tailed *t*-test, *p* > 0.22).

To enhance the saliency of the feedback, only the highest rated profiles were shown to the participants during the fMRI scan. Participants were reminded at the start of the fMRI scan that they would only see profiles that were highly rated by them. As in our previous studies, the SFT does not involve deception, however, participants were asked to immerse themselves in the experience and respond as if the feedback was real, resulting in significant behavioral and neural responses (Hsu et al., 2013; Yttredahl et al., 2018). Inside the scanner, participants viewed a picture of themselves (500 ms) along with a picture of a highly rated profile (500 ms) followed by one of three types of feedback (4 s): acceptance (Acc), rejection (Rej), and neutral (Neu) in a block design (Figure 1B; Yttredahl et al., 2018). Each feedback type was presented in blocks consisting of four trials. fMRI images were collected in four runs, with each run consisting of six pseudorandomized blocks. Each run lasted 3 min and 12 s plus approximately 30 s shim times between runs.

During the screening visit, participants completed questionnaires that measure affect and motivation-related traits. In our previous study (Yttredahl et al., 2018), we found that left and right NAcc mediate trait reward responsiveness and increased ratings of feeling “happy and accepted” following acceptance in HCs, but not in MDD patients.

#### Emotion Ratings

Changes in emotional states in response to the SFT were measured in a separate testing session outside of the MRI scanner, since performing subjective ratings of emotionally salient stimuli has been shown to attenuate activity in areas such as the AI and amygdala (Taylor et al., 2003). Participants viewed a variant of the SFT whereby they were shown a block of 18 trials of each feedback type and were asked to indicate changes in emotional states following each block (Yttredahl et al., 2018). Responses were recorded on a 5-point Likert-type scale using a button-press response box. Similar to our previous studies (Hsu et al., 2013, 2015), for each participant scores for “sad” and “rejected” were averaged, and “happy” and “accepted” were averaged. These averaged scores were correlated with neural activations in the NAcc and AI during social acceptance and social rejection.

#### fMRI Acquisition

Functional image volumes (BOLD signal) were obtained using a T2^*^-weighted pulse sequence on a 3.0 Tesla GE Sigma 9.0 scanner (Milwaukee, WI, USA) with a standard radiofrequency coil at the University of Michigan, Ann Arbor, MI, USA. Images were acquired using a single-shot combined spiral in/out sequence to reduce signal dropout in subcortical areas and around sinus regions. For each volume, 29 slices were acquired using the following parameters: repetition time, TR: 2,000 ms; echo time, TE: 30 ms; flip angle: 90°; field of view, FoV: 20 cm × 20 cm, 64 × 64 matrix; in-plane resolution: 3.13 × 3.13 mm; slice thickness: 4 mm.

A high-resolution T1-weighted pulse sequence provided anatomical localization (3D spoiled gradient recalled echo; TR, 12 ms; TE, 5 ms; TI, 500 ms; flip angle, 15°; FoV, 26 cm × 26 cm, 256 × 256 matrix; in-plane resolution, 1.02 × 1.02 mm; slice thickness, 1.2 mm).

#### fMRI Image Analysis

Functional images were preprocessed using a standard pipeline in FMRIB Software Library (FSL). Images were slice-time corrected and realigned to correct for motion artefacts. Images were reviewed for head movement >3 mm translation or 3° rotation. All 40 participants were included in the analysis of the MID (i.e., 20 MDD and 20 HCs). SFT data from one MDD patient was excluded from further analyses due to broad signal dropout in the striatum across all runs of the SFT (Yttredahl et al., 2018). SFT data from one HC were excluded due to excessive movement beyond our specified threshold of movement ≥3 mm maximum displacement (x, y or z direction) or ≥3 degrees of angular motion. Thus, the final sample for the SFT consisted of 19 MDD patients and 19 HCs. The six motion parameters were added as nuisance regressors to our fMRI model.

Using FSL, high-resolution T1 images were co-registered to the participants’ functional images, segmented into tissue probability maps, and normalized to Standard Montreal Neurological Institute (MNI) space. The functional images were normalized using FSL and smoothed (5 mm full-width at half maximum) using a Gaussian Kernel.

First-level analysis was performed in Statistical Parametric Mapping v.8 (SPM8; Wellcome Institute of Cognitive Neurology, London, UK) using the General Linear Model (GLM), and maps were created for the primary contrasts of interest. The primary contrasts of interest were CW-Neu and CL-Neu, however, an exploratory analysis examined the neural response to the cue phase for “uncertain” wins and losses (i.e., UW-Neu and UL-Neu; Supplementary Tables S2, S3). For the SFT task, the primary contrasts of interest were Acc-Neu and Rej-Neu. Based on our hypothesis, we examined activations in the NAcc and AI during responses to both positive and negative monetary and social incentive stimuli. ROIs were anatomically defined using the Harvard Brain Atlas probability masks, thresholded at 0.25 confidence, and binarized (Yttredahl et al., 2018). The AI masks were bounded posteriorly at *y* = 8, based on a previous study that investigated neural responses to social exclusion (Way et al., 2009). A single mask comprising bilateral AI and bilateral NAcc was used for analysis in SPM8. All ROI analyses reported herein used an initial height threshold of *p*_uncorrected_ < 0.001 (*k* > 10), and subsequent small volume correction in *a priori* ROIs [SVC using family wise-error correction (FWE)] at *p*_FWE-SVC_ < 0.05).

#### Conjunction Analysis

To test our hypothesis that AI and NAcc were associated with salience of the stimuli regardless of valence, we performed a logical “AND” conjunction analysis (Subramaniam et al., 2015, 2016) to determine if there were voxels within the individual ROIs that were common to both certain wins and certain losses, as well as social acceptance and rejection. ROIs were chosen for conjunction only if both contrasts within the same task (i.e., CW-Neu and CL-Neu or Acc-Neu and Rej-Neu) showed activations either during within-group or between-group analyses.

Using Imcalc in SPM8, SVC thresholded maps (*p*_FWE-SVC_ < 0.05) were binarized for each condition. The binarized images were used to produce conjunction maps using the equation: i1 + (2 * i2) (Subramaniam et al., 2015).

## Results

### Behavior

#### MID Task

A repeated measures ANOVA showed a significant main effect of task condition on hit rate (*F*_(3.52, 133.91)_ = 10.04, *p* < 0.001) as well as reaction time (*F*_(4,152)_ = 15.79, *p* < 0.001) across all participants.

We did not find a significant group × condition interaction either for hit rate (*F*_(3.52,133.91)_ = 0.31, *p* = 0.85) or for reaction time (*F*_(4,152)_ = 0.28, *p* = 0.89) suggesting that the MDD group did not differ from the HCs in their performance on the MID. Additional analyses of behavioral data for the MID are reported in Supplementary Table S1.

#### SFT

MDD patients showed enhanced behavioral responses to social acceptance as well as social rejection compared to HCs, as shown by significantly greater increases in feeling “happy and accepted” during social acceptance (*t*_(36)_ = 2.03, *p* = 0.05), as well as a trend for greater decreases in feeling “happy and accepted” during social rejection (*t*_(30)_ = 1.65, *p* = 0.11). In addition, MDD patients also exhibited significantly increased desire to socialize (*t*_(27.16)_ = 3.06, *p* = 0.005), and decreased “sad and rejected” (*t*_(27.15)_ = 2.64, *p* = 0.01), as well as a trend for significant increases in self-esteem (*t*_(21.37)_ = 1.81, *p* = 0.09) during social acceptance compared with HCs.

### Functional MRI

#### MID Task

**Figure 2:**
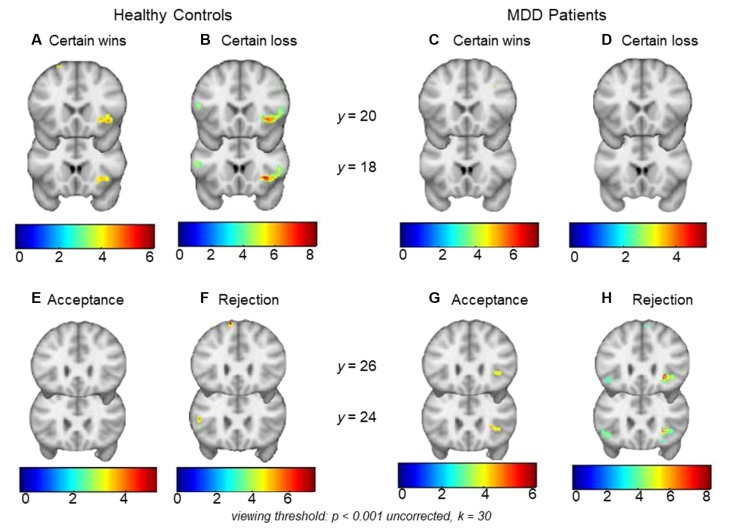
Top row: panels **(A)** and **(B)** show right anterior insula (AI) activations during monetary certain wins and losses (minus neutral) respectively in healthy controls (HCs). Within-group analysis showed significant activations during monetary wins and loss in the right AI. Within-group analysis in major depressive disorder (MDD) did not show significant right AI activations either during **(C)** monetary wins or during **(D)** monetary loss (minus neutral). Bottom row: panels **(E)** and** (F)** show an absence of activation in HCs during social acceptance and rejection (minus neutral), whereas **(G)** and **(H)** show right AI activations during social acceptance and social rejection (minus neutral) in MDD patients. Within-group analysis showed significant activations during social rejection and a trend for significant activations during acceptance in the right AI. Viewing threshold: *p* < 0.001 (uncorrected), *k* = 30. Color bars represent range of *t*-values. Coronal sections of the brain are presented with the montreal neurological institute (MNI) *y* coordinates.

HCs showed significant right AI activations during monetary wins (*x, y, z* = 40, 20, −6, *t* = 4.92, *k* = 74, *p*_FWE-SVC_ = 0.027) as well as losses (*x, y, z* = 30, 12, −8, *t =* 8.29, *k* = 144, *p*_FWE-SVC_ < 0.001; Figure 2). In MDD patients, significant activations were not found within the *a priori* regions AI or NAcc for certain wins or certain losses (both *p*_FWE-SVC_ > 0.05). Between-group analyses did not reveal significant differences between MDD patients and HCs during anticipation of either wins (CW-Neu) or losses (CL-Neu).

**Figure 3:**
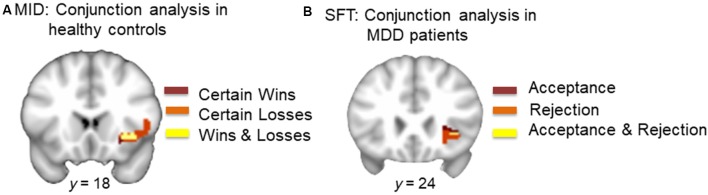
**(A)** Conjunction analysis of monetary wins and losses in HCs. Dark red represents voxels in the right AI during certain wins (minus neutral). Orange represents voxels in the right AI during certain loss (minus neutral). Yellow represents overlapping voxels in the AI during both win and loss (center of mass: *x* = 36.6, *y* = 21.5, *z* = −3.8, *k* = 126). **(B)** Conjunction analysis of acceptance and rejection in MDD patients. Dark red represents voxels in the right AI during acceptance (minus neutral). Orange represents voxels in the right AI during rejection (minus neutral). Yellow represents overlapping voxels in the AI during both social acceptance and social rejection (center of mass: *x* = 35.6, *y* = 24.4, *z* = −2.0, *k* = 5).

Conjunction analysis of monetary gain (CW-NT) and monetary loss (CL-NT; individual SVC thresholded maps) in HCs revealed overlapping voxels in the right AI (center of mass: *x, y, z* = 36.6, 21.5, −3.8; *k* = 126; Figure 3A).

#### SFT

MDD patients showed significant activations in the right AI during social rejection (*x, y, z* = 28, 24, −4, *t* = 8.57, *k* = 26, *p*_FWE-SVC_ < 0.001), and a trend for significance in the same region during social acceptance (*x, y, z* = 28, 24, 2, *t* = 4.66, *k* = 16, *p*_FWE-SVC_ = 0.051; Figure 2). In HCs, significant activations were not found within the *a priori* regions AI or NAcc during acceptance or rejection trials (both *p*_FWE-SVC_ > 0.05). Between-group analyses did not reveal significant differences between MDD patients and HCs either during social acceptance (Acc-Neu) or during social rejection (Rej-Neu; both *p*_FWE-SVC_ > 0.05). Parameter estimates for the right AI during social acceptance and rejection were extracted in MDD patients. We did not find significant associations between neural activations and emotional rating scores (*p*’s > 0.05).

Conjunction analysis of acceptance (Acc-Neu) and rejection (Rej-Neu; individual SVC thresholded maps) in MDD patients revealed overlapping voxels in the right AI (center of mass: *x, y, z* = 35.6, 24.4, −2.0; *k* = 5; Figure 3B).

## Discussion

The aim of the present study was to examine salience-related neural representation of monetary and social reward and loss in women with a diagnosis of MDD compared to HC women. Several studies have compared responses to monetary vs. social stimuli in HCs (Izuma et al., 2008; Rademacher et al., 2010; Lin et al., 2011; Xie et al., 2014), however, no study to our knowledge has examined these responses in MDD patients. Investigating how salience-related neural responses are differently represented in MDD based on the type of the incentive stimuli is important for improving our understanding of the nature, function, and clinical implications of reward-related abnormalities in MDD.

Our results highlight two important findings. First, the within-group analysis showed that in response to monetary stimuli, HCs but not MDD patients showed significant activations in the right AI during both monetary gain and monetary loss. Conjunction analysis further showed that monetary gain and loss activated overlapping voxels within the right AI (Figure 3A). Second, patients with MDD, but not HCs showed a trend for significant activations in the right AI during social acceptance as well as strong activations in the same region during social rejection, in the within-group analysis. Conjunction analysis showed overlapping voxels within the right AI that responded to both acceptance and rejection (Figure 3B). Although direct comparisons between HCs and MDD patients in response to the MID or SFT were not significant, the within-group and conjunction analyses suggest a dissociable processing of reward and loss in MDD vs. HCs. This highlights the critical need to assess neural responses to both positive and negative stimuli, especially in the social domain in MDD. This differential response is particularly notable given that participants were informed that monetary incentives were real (i.e., participants were paid for money won during the MID), whereas the social feedback was only simulated (i.e., no deception was involved), suggesting that MDD patients experienced real monetary incentives as less salient than simulated social feedback.

The AI plays a key role in diverse functions and behaviors such as interoception, attention, and saliency, *via* projections to the NAcc and reciprocal connections with limbic and reward-related brain regions such as the amygdala, anterior and middle cingulate and the orbitofrontal cortex (Allen et al., 1991; Flynn, 1999; Rolls, 2016). In the social context, the AI is activated to understand the feelings of others (Lamm and Singer, 2010), and in response to both social inclusion and exclusion (Dalgleish et al., 2017). In patients with MDD relative to HCs, greater activations in the AI were found in response to both positive and control feedback conditions (Davey et al., 2011) as well as social exclusion (Kumar et al., 2017). In accordance with these studies, we found heightened AI activations in response to both social acceptance and rejection in MDD, which was not found in HCs. Overall, our results are supported by studies suggesting a more general role for the AI in salience processing during social feedback (Dalgleish et al., 2017) especially feedback directed at the self vs. others (Perini et al., 2018), rather than the valence of feedback.

A role for the AI in salience processing is not restricted to social stimuli. A number of neuroimaging studies have demonstrated a role for the AI during anticipation of aversive imagery, anticipation and experience of painful stimuli, and the encoding of monetary loss (Ploghaus et al., 1999; O’Doherty J. et al., 2003; Simmons et al., 2004; Koyama et al., 2005). However, the AI also responds to rewarding stimuli (Jessup and O’Doherty, 2014) suggesting that the salience of the outcome, and not only the valence drives activation in this region. As a key node of the salience network, AI initiates signals to engage higher order brain regions important for attentional processing and cognitive control (Menon and Uddin, 2010). In addition to assigning significance to the external stimuli, the AI is important for perception of internal bodily states (Craig, 2002, 2009; Critchley et al., 2004) and activations are found to correlate with participants’ subjective emotional experiences (Zaki et al., 2012), suggesting an association between AI activations, subjective states, and experience of emotions (Critchley et al., 2004). In the present study, AI activations in MDD patients likely reflect the detection of salient social information, rather than interoception-related activations, since AI activations and subjective emotional experiences to feedback were not significantly correlated in this study.

Our results indicate that social feedback is a salient event that elicits a greater response in MDD patients. In HCs, AI activations in response to monetary stimuli are consistent with its role in the anticipation of a salient outcome and might suggest increased sensitivity to monetary cues, a finding absent in MDD patients. The increased AI activation in HCs in the present study in response monetary reward and loss cues is consistent with previous meta-analyses in HCs showing increased engagement in the AI during both reward and loss anticipation (Oldham et al., 2018). Whether these dissociable responses to social and monetary incentive stimuli are also found in men will need to be ascertained in future studies.

Optimal activity in the AI is crucial to initiate appropriate responses to salient events (Uddin and Menon, 2009). Negative emotional stimuli (Hamilton et al., 2012) and to some extent positive and neutral stimuli (Davey et al., 2011) are shown to elicit greater AI activations in MDD patients vs. HCs, suggesting an over-reactive salience detection system. Not surprisingly, enhanced baseline activation in the AI in MDD is also predictive of poor response to any subsequent treatment (Fu et al., 2013). Whether AI in our MDD patients represent a pathological response to social feedback and the mechanisms by which this response contributes to dysfunctional reward and loss processing need to be ascertained in future studies.

We did not find significant differences in NAcc activations between MDD patients and HCs in either the MID or SFT. The NAcc has been shown to be more strongly recruited during the anticipation of rewards (Knutson et al., 2001; Ernst et al., 2004; Carter et al., 2009) and losses (Carter et al., 2009) compared to reinforcing outcomes (Knutson et al., 2001; Ernst et al., 2004), indicating that activity in this region may be more strongly activated by uncertain or unpredictable events. In support, several studies showed the association between NAcc and prediction error (Pagnoni et al., 2002; O’Doherty J. P. et al., 2003; Rodriguez et al., 2006), and that uncertainty of both wins, as well as losses, engaged the NAcc (Cooper and Knutson, 2008). The lack of significant activations in the NAcc may reflect limited uncertainty in the MID and SFT, since we modeled expected wins and losses in the MID, and the SFT only models expected outcome of acceptance or rejection feedback. Exploratory analyses showed that both uncertain wins, as well as uncertain losses, activated NAcc in both HCs and MDD patients (Supplementary Material, Results), confirming that uncertainty of monetary wins and losses more strongly activate the NAcc.

The majority of MDD patients (*n* = 17) in our sample exhibited sub-threshold symptoms of anxiety, and one met DSM-IV criteria for social anxiety disorder, raising the possibility that anxiety may have contributed to our findings. Previous studies have shown heightened striatal activation in response to unexpected positive feedback in socially anxious adolescents (Jarcho et al., 2015), or striatal hypersensitivity to increasing magnitudes of monetary gains or losses in adolescents with social phobia (Guyer et al., 2012). However, it is unclear how these findings relate to the present study, given our focus on adults with a primary diagnosis of MDD, and our findings in the AI but not the striatum. Nevertheless, future studies will need to examine differential responses to monetary and social incentives in MDD with and without comorbid social phobia.

Several limitations should be noted. First, the MID is designed as an event-related fMRI task that examines neural activation during *anticipation* of expected reward or loss, whereas the SFT is a blocked design that examined neural activation during the *consummatory* experience of social acceptance or rejection. Thus, it is possible that while MDD patients showed deficits in the anticipation of reward or loss, this may also occur in a task examining the *anticipation* of acceptance and rejection. A previous study showed that the anticipation of monetary and social rewards activated similar brain regions whereas the consumption of monetary and social rewards activated different areas (Rademacher et al., 2010), however, another study found that the consumption of monetary and social rewards activated similar areas (Wake and Izuma, 2017). Together these studies provide partial support that anticipation and consumption of monetary rewards activate similar areas. More fine-grained studies will need to examine both the anticipatory and consummatory phases of monetary vs. social reward in identical tasks, in both MDD patients and HCs. Second, our modest sample sizes may have led to insufficient power to detect significant between-group effects. Future studies will need to examine sex differences in AI activation during acceptance and rejection in MDD patients vs. HCs. Third, emotion ratings in response to the SFT were assessed after the scan, which may not be the most accurate representation of emotional responses during the scan. Fourth, connectivity analyses may have provided additional information on regional networks that may be altered across social and non-social contexts in MDD.

In conclusion, we present preliminary evidence for dissociable neural responses to monetary and social stimuli in HC and MDD women. In response to the MID, HCs but not patients with MDD showed AI activations during both monetary reward and loss. In response to the SFT, MDD patients but not HCs showed AI activations during both social acceptance and rejection. The common neural responses in the AI across both positive and negative stimuli may indicate activations associated with the detection of salient information regardless of valence. Importantly, these findings highlight differential neural representations of salience to monetary and social domains as a function of MDD diagnosis in women, suggesting that future investigations of reward and loss systems in MDD need to consider both domains.

## Data Availability

The datasets generated for this study are available on request to the corresponding author.

## Ethics Statement

This study was carried out in accordance with the recommendations of the University of Michigan Medical School Institutional Review Board, with written informed consent from all subjects. All subjects gave written informed consent in accordance with the Declaration of Helsinki. The protocol was approved by University of Michigan Medical School Institutional Review Board.

## Author Contributions

AS analyzed the data, interpreted results, and wrote the manuscript. AY and EF assisted with data analyses and interpretation. BM, TL, and SL assisted with research design, data analyses and interpretation, and manuscript edits. DH designed and conducted the study, and assisted with data analysis, interpretation, and manuscript edits.

## Conflict of Interest Statement

BM has acted as a consultant to Alkermes and has received research funds from Novartis for work unrelated to this manuscript. The remaining authors declare that the research was conducted in the absence of any commercial or financial relationships that could be construed as a potential conflict of interest. The reviewer JLW declared a shared affiliation, with no collaboration, with one of the authors, DH, to the handling editor at time of review.
