# Supplementary material for: Dissociable Neural Responses to Monetary and Social Gain and Loss in Women With Major Depressive Disorder
Source: Front Behav Neurosci. 2019 Jul 11;13:149. doi: 10.3389/fnbeh.2019.00149 (PMC6637282; doi:10.3389/fnbeh.2019.00149)
Supplement: Supplementary file 1 [file Table_1.docx]

**Supplementary Results**

# **Behavior**

Post-hoc tests were performed following analyses with repeated measures ANOVA (results detailed in the main text). *T* tests on all participants confirmed that average hit rate was higher during certain wins compared to neutral condition (*t*_39_ = 3.78, *p* = 0.001). For the uncertain condition, all participants had higher hit rate as well as quicker response time during uncertain wins as well as uncertain loss compared to neutral condition (*t*_39_ > 4.71, p<0.001). Although we varied the target duration to ensure an average hit rate of 60% for each participant across the task, the algorithm did not modulate presentation time for each condition type separately.

There was no effect of run order on hit rates (*t*_39_ = 0.75, *p* = 0.46), however, we found an effect of run order on reaction time whereby all participants responded faster on the second MID run (*t*_39_ = 2.23, *p* = 0.031), consistent with a previous study (Mickey et al., 2016).

**Table S1: Post-hoc behavioral results for the MID task**

| \| Condition \| *t* \| *p* \| \| --- \| --- \| --- \| \| Hit Rate \|  \|  \| \| CW>CL \| 2.05 \| 0.047 \| \| CW>Neu \| 3.78 \| 0.001 \| \| UW>Neu \| 4.71 \| <0.001 \| \| UL>Neu \| 5.71 \| <0.001 \| \|  \|  \|  \| \| Reaction Time \| \|  \| \| UW>Neu \| 4.95 \| <0.001 \| \| UL>Neu \| 7.50 \| <0.001 \| |  |  |
| --- | --- | --- | --- | --- | --- | --- | --- | --- | --- | --- | --- | --- | --- | --- | --- | --- | --- | --- | --- | --- | --- | --- | --- | --- | --- | --- | --- | --- | --- | --- | --- | --- |
|  |  |  |

# **Functional MRI**

***Uncertain Wins – Neutral:*** In patients with MDD, we found enhanced activations during uncertain wins (minus neutral) in the bilateral nucleus accumbens and anterior insula. In healthy controls, activations were limited to the bilateral nucleus accumbens during uncertain wins (**Table S2**). We did not find significant differences between MDD patients and healthy controls during uncertain loss in our *a priori* regions of interest.

**Table S2: Within group analyses in MDD patients and healthy controls during uncertain wins**

| Region | *x* | *y* | *z* | *K* | *t* | *p*_FWE-SVC_ |
| --- | --- | --- | --- | --- | --- | --- |
| MDD patients |  |  |  |  |  |  |
| *UW-Neu* |  |  |  |  |  |  |
| Left NAcc | -14 | 16 | -4 | 55 | 5.36 | 0.012 |
| Right NAcc | 14 | 14 | -8 | 53 | 5.05 | 0.020 |
| Right AI | 30 | 24 | 2 | 60 | 5.03 | 0.021 |
| Left AI | -34 | 16 | -4 | 33 | 4.66 | 0.041 |
| *Neu-UW* |  |  |  |  |  |  |
|  | - | - | - | - | - | ns |
| Healthy controls |  |  |  |  |  |  |
| *UW-Neu* |  |  |  |  |  |  |
| Left NAcc | 0 | -8 | -2 | 14 | 5.64 | 0.007 |
| Right NAcc | 0 | 10 | -4 | 20 | 5.37 | 0.012 |
| *Neu-UW* |  |  |  |  |  |  |
|  | - | - | - | - | - | ns |

Abbreviations: UW-Neu: uncertain wins (minus neutral); Neu-UW: neutral (minus uncertain wins). NAcc: nucleus accumbens; AI: anterior insula; ns: non-significant. Results reported herein used an initial height threshold of *p*_uncorrected_ < 0.001 (*k* > 10) and subsequent small volume correction in *a priori* regions of interest (SVC using family wise-error correction [FWE]) at *p*_FWE-SVC_ < 0.05).

***Uncertain Loss – Neutral:*** Uncertain loss (minus neutral) was associated with increased activations in the bilateral nucleus accumbens in MDD patients. In healthy controls, uncertain loss was associated with increased activations in the left NAcc as well as the bilateral AI (**Table S3**). Between-group analyses did not reveal significant differences between MDD patients and healthy controls in our *a priori* regions of interest.

**Table S3: Within group analyses in MDD patients and healthy controls during uncertain loss**

| Region | | *x* | *y* | *z* | *K* | *t* | *p*_FWE-SVC_ |
| --- | --- | --- | --- | --- | --- | --- | --- |
| MDD patients |  | |  |  |  |  |  |
| *UL-Neu* | |  |  |  |  |  |  |
| Right NAcc | | 14 | 14 | -6 | 28 | 5.43 | 0.014 |
| Left NAcc | | -8 | 14 | -2 | 26 | 5.35 | 0.016 |
| *Neu-UL* | |  |  |  |  |  |  |
|  | | - | - | - | - | - | ns |
| Healthy controls | |  |  |  |  |  |  |
| *UL-Neu* | |  |  |  |  |  |  |
| Right AI | | 40 | 20 | -2 | 136 | 6.29 | 0.003 |
| Left AI | | -40 | 16 | -6 | 168 | 5.95 | 0.005 |
| Left NAcc | | -8 | 12 | -2 | 17 | 5.07 | 0.025 |
| *Neu-UL* | |  |  |  |  |  |  |
|  | | - | - | - | - | - | ns |

Abbreviations: UL-Neu: uncertain loss (minus neutral); Neu-UL: neutral (minus uncertain loss).; NAcc: nucleus accumbens; AI: anterior insula; ns: non-significant. Results reported herein used an initial height threshold of *p*_uncorrected_ < 0.001 (*k* > 10) and subsequent small volume correction in *a priori* regions of interest (SVC using family wise-error correction [FWE]) at *p*_FWE-SVC_ < 0.05).

The NAcc has been shown to be more strongly recruited during the anticipation of rewards (Carter et al., 2009; Ernst et al., 2004; Knutson et al., 2001) and losses (Carter et al., 2009) compared to reinforcing outcomes (Ernst et al., 2004; Knutson et al., 2001). The uncertainty of both wins as well as losses is shown to engage the NAcc (Cooper and Knutson, 2008). Consistent with this, findings from our exploratory within-group analyses show that both uncertain wins as well as uncertain losses activated NAcc in both HCs and MDD patients (**Table S2 and S3**).

Uncertain outcomes are associated with high salience (Cooper and Knutson, 2008; Mickey et al., 2016). In addition to NAcc activation in MDD and HCs during both uncertain win and loss, within-group analyses found that MDD patients showed additional increases in bilateral AI activations in response to uncertain wins **(Table S2),** whilst HCs showed bilateral AI activations in response to uncertain loss (**Table S3**). There were no significant differences between MDD patients and HCs during either uncertain win or loss. Prior research using the MID task, in which cues indicate potential reward and loss (uncertain outcomes), found that anticipation of both reward and loss engaged the AI in MDD patients as well as in HCs (Knutson et al., 2008; Oldham et al., 2018; Smoski et al., 2011). Findings from the within-group analysis in the present study indicate a differential neural responsivity in the AI in MDD and HCs to anticipation of uncertain reward and loss, however they must be considered with caution in the absence of previous studies that show similar dissociable responses in MDD and HCs to anticipation of uncertain incentive cues.

# **References**

Carter, R. M., MacInnes, J. J., Huettel, S. A., and Adcock, R. A. (2009). Activation in the VTA and nucleus accumbens increases in anticipation of both gains and losses. *Frontiers in behavioral neuroscience*.

Cooper, J. C., and Knutson, B. (2008). Valence and salience contribute to nucleus accumbens activation. *Neuroimage* 39, 538–547.

Ernst, M., Nelson, E. E., McClure, E. B., Monk, C. S., Munson, S., Eshel, N., et al. (2004). Choice selection and reward anticipation: an fMRI study. *Neuropsychologia* 42, 1585–1597.

Knutson, B., Bhanji, J. P., Cooney, R. E., Atlas, L. Y., and Gotlib, I. H. (2008). Neural responses to monetary incentives in major depression. *Biological psychiatry* 63, 686–692.

Knutson, B., Fong, G. W., Adams, C. M., Varner, J. L., and Hommer, D. (2001). Dissociation of reward anticipation and outcome with event-related fMRI. *Neuroreport* 12, 3683–3687.

Mickey, B. J., Heffernan, J., Heisel, C., Peciña, M., Hsu, D. T., Zubieta, J.-K., et al. (2016). Oxytocin modulates hemodynamic responses to monetary incentives in humans. *Psychopharmacology* 233, 3905–3919.

Oldham, S., Murawski, C., Fornito, A., Youssef, G., Yücel, M., and Lorenzetti, V. (2018). The anticipation and outcome phases of reward and loss processing: A neuroimaging meta‐analysis of the monetary incentive delay task. *Human brain mapping* 39, 3398–3418.

Smoski, M. J., Rittenberg, A., and Dichter, G. S. (2011). Major depressive disorder is characterized by greater reward network activation to monetary than pleasant image rewards. *Psychiatry Research: Neuroimaging* 194, 263–270.
